# Frequency, characteristics and hospital outcomes of road traffic accidents and their victims in Guinea: a three-year retrospective study from 2015 to 2017

**DOI:** 10.1186/s12889-019-7341-9

**Published:** 2019-07-31

**Authors:** Karifa Kourouma, Alexandre Delamou, Léopold Lamah, Bienvenu Salim Camara, Delphin Kolie, Sidikiba Sidibé, Abdoul Habib Béavogui, Philip Owiti, Marcel Manzi, Serge Ade, Anthony D. Harries

**Affiliations:** 1 Centre National de Formation et de Recherche en Santé Rurale (CNFRSR) de Maferinyah, PB: 4099, Maferinyah, Forécariah, Guinea; 2 Department of Public Health, Gamal Abdel Nasser University of Conakry, Conakry, Guinea; 3 Department of Traumatology and Orthopedics, University Teaching Hospital of Donka, Conakry, Guinea; 4 International Union Against Tuberculosis and Lung Disease, Paris, France; 5 The National Tuberculosis, Leprosy and Lung Disease Program, Ministry of Health, Nairobi, Kenya; 6 Medical Department, Médecins Sans Frontière Bruxelles, Bruxelles, Belgium; 7 Faculté de Médecine, Université de Parakou, Parakou, Benin; 8 London School of Hygiene and Tropical Medicine, London, UK

**Keywords:** Road traffic accident, Injuries, Death, Guinea, Police station, Hostpital, SORT IT

## Abstract

**Background:**

Road traffic accidents (RTA) remain a global public health concern in developing countries. The aim of the study was to document the frequency, characteristics and hospital outcomes of road traffic accidents in Guinea from 2015 to 2017.

**Methods:**

We conducted a retrospective cohort study using medical records of RTA victims from 20 hospitals and a cross-sectional study of RTA cases from eight police stations in eight districts in Guinea, West Africa. Data analysis included descriptive statistics, trends of RTA, a sequence of interrupted time-series models and a segmented ordinary least-squares (OLS) regression.

**Results:**

Police stations recorded 3,140 RTA over 3 years with an overall annual increase in RTA rates from 14.0 per 100,000 population in 2015, to 19.2 per 100,000 population in 2016 (37.1% annual increase), to 28.7 per 100,000 population in 2017 (49.5% annual increase).

Overall, the injury rates in 2016 and 2017 were .05 per 100,000 population higher on average per month (95% CI: .03–.07). Deaths from RTA showed no statistical differences over the 3 years and no association of RTA trends with season was found.

Overall, 27,751 RTA victims were admitted to emergency units, representing 22% of all hospitals admissions. Most victims were males (71%) and young (33%). Deaths represented 1.4% of all RTA victims. 90% of deaths occurred before or within 24 h of hospital admission. Factors associated with death were being male (*p* = .04), being a child under 15 years (*p* = .045) or an elderly person aged ≥65 years (*p* < .001), and having head injury or coma (*p* < .001).

**Conclusions:**

RTA rates in Guinea are increasing. There is a need for implementing multisectoral RTA prevention measures in Guinea.

## Background

Road traffic accidents (RTA) represent a huge global public health concern due to their increasing occurrence, related deaths and disabilities, social and financial consequences [1]. In 2018, the World Health Organization (WHO) estimated that 1.35 million people globally die each year from RTA [1, 2], and this is alongside the additional 20–50 million people who are seriously injured or disabled [3]. Low and Middle-Income countries, which house more than half (60%) of the world’s motor vehicles, bear more than 90% of RTA deaths and associated morbidities [1]. This growing public health problem has led to one of the health-related Sustainable Development Goals (SDGs) being focused on RTA with a target to halve the global number of accidents by 2020 [4]. This will be a major challenge because without effective interventions and action on the ground, RTA are estimated to increase by 67%, and become the fifth leading cause of death globally by 2030 [5].

In Sub-Saharan Africa, RTA have increased from 41 to 93 per 100,000 population over a fifteen-year period up to 2015, and currently account for one third of trauma cases admitted to health facilities [6, 7]. RTA case fatality rates for the Africa region in 2013 were estimated at 27 per 100,000 population [8]. Survivors of RTA are additionally burdened with physical disabilities and psychological disorders, especially affecting young adults, and the consequences of RTA also extend beyond the victims to the families and the associated communities. This high burden of RTA and associated deaths is due to a number of factors including inadequate or weakly enforced road safety legislation or ignorance of this legislation by road users, bad road infrastructure, irregular or non-existent vehicle maintenance and poor access to good quality health care [9].

Various studies in Africa have reported on RTA mortality rates ranging from 0.3 to 41% and occurring among car drivers, passengers, pedestrians, cyclists or users of motorcycles, with victims being predominantly males and in the age group of 20 to 44 years [6, 7]. In Nigeria, the most populated country in the continent, the trend in RTA has been reported to be increasing and RTA represent the leading cause of injury related deaths and the most common cause of disability in the country [10]. Recently in Ethiopia, similar findings reported that only one third of RTA victims received urgent/immediate surgical intervention before admission to hospital [11].

In Guinea, West Africa, the number of motor vehicles and motorcycles have increased from approximately 16,000 in 2010 to 37,000 in 2015 [12]. In line with other countries in the region, RTA have also been reported to be increasing in recent years [12], with RTA-related death rates estimated at 10–20 per 100,000 population [13, 14]. In terms of morbidity there has only been one publication from Guinea which focused solely on brain injury [15]. Given the rising importance of this public health problem, it is crucial for Guinea to assess the burden, characteristics and hospital outcomes of RTA, and especially to have baseline data by which to monitor the RTA-related SDG targets.

The study aimed therefore to document the frequency, characteristics and hospital outcomes of RTA in Guinea from 2015 to 2017 by using two data sources (police station and hospital records).

## Methods

### Study design

This study combined a retrospective cohort study using routinely collected data of RTA victims admitted to 20 hospitals from eight health districts and a cross-sectional study of RTA cases from eight police stations from the same districts.

### Setting

#### General setting

Guinea, located in West Africa, is one of the low-income countries in the world with 55% of its population living below the national poverty line [12]. According to the Health Management and Information System data published in 2016, there were more than 11 million inhabitants with the majority living in rural settings (71%) and over half being illiterate (67%) [12].

The country comprises eight administrative regions including the capital city Conakry and 33 districts. The national public health system is tiered into a primary level (413 rural and urban health centers), a secondary level (7 regional and 26 district hospitals, 8 communal health centers with five in Conakry), and a tertiary level (3 national hospitals) [12]. There are 33 Medical and Surgical clinics and 11 polyclinics in the country. In Guinea, the total length of the road network is about 44,000 km of which only 2,220 is paved with asphalt nationwide [12].

#### Site specific setting

In the eight urban districts including the capital city (Conakry, Boké, Kindia, Kankan, N’Zérékoré, Mamou, Faranah and Siguiri), there have been reports of a high burden of RTA from 2014 to 2016 [12]. These districts constituted our study sites in which approximately 4.71 million people live (45% of the national population). Only one district (the capital city) out of the eight has a low proportion of its population living beneath the national poverty line (27.4 to 37.3%) [12].

#### General management of RTA

There are two police stations in Conakry and one in each of the seven urban districts which have a register in which to record details of all RTA. Generally, all RTA should be reported to the nearest police station, following which the police makes a report and enters specific data into the police register on: date of RTA, vehicle types, numbers of persons involved in the RTA, injuries (minor or severe) and death. Persons with no injury are sent home whereas those with injuries and the dead bodies are sent to the nearest hospital emergency unit. Alternatively, some RTA victims make their own way to a hospital without involving the police stations. At hospital, the injured victims are clinically assessed by trauma specialists or general surgeons according to the type of hospital (tertiary or secondary) and a record made in case files and registers of socio-demographic and clinical characteristics, types of injury sustained and death that occurred before or on arrival. Therefore, information collected at police stations fit the pre-crash and crash phase of Haddon Matrix while those of hospitals correspond to the post-crash phase [16].

Furthermore, the injured victims, according to their clinical status and Glasgow Coma Score (GCS) that had been adapted for Guinea [17], can stay in the emergency unit for up to 24 h at which time they are either discharged home or admitted to an appropriate departmental ward (surgical, orthopedic or intensive care unit). The emergency unit case files of those admitted are transferred to the wards, and in these case files and in the ward registers, a record is made of types of intervention, duration of hospital stay and discharge outcome including death. In addition, police stations record all reported RTA cases along with number of victims, with or without injury and death, and hospitals also record details of all admitted RTA victims even those with no injuries.

#### Emergency units and admission to hospitals departments

In Conakry, the public health hospitals included the three national tertiary hospitals and five communal medical centers, all of which have an emergency unit. The two frequently used private hospitals and one private clinic, which all had an emergency unit, were also included, giving a total of 11 hospitals in Conakry. In each of the seven urban districts, the referral district hospital and two private clinics that had an emergency unit were included, giving a total of 20 hospitals.

### Study population

All RTA and all persons involved in RTA registered at the selected police stations and public/private hospitals in eight urban districts of Guinea between January 2015 and December 2017 were included in the study.

### Data variables, sources of data and data collection

Police station data variables included: month and year of RTA; type of RTA (motor vehicle, motorcycle, bicycle, motor vehicle + motor vehicle, motor vehicle + motorcycle, motor vehicle + bicycle, motor vehicle + pedestrian, motorcycle + motorcycle, motorcycle + bicycle, motorcycle + pedestrian); persons involved in the RTA along with injury (minor or severe) and death. Data were collected from RTA registers at the eight selected police stations. Data from one police station were not included because these were raw annual aggregated data rather daily or monthly.

Hospital data variables included: age, sex, occupation, type of road user, month and year of admission to the emergency unit, type of hospital (public or private), predominant type and anatomical location of the injury, GCS (Normal = 15; Mild coma = 14–10; Heavy coma = 9–7; Deep coma = 6–3), interventions, hospital departmental wards, hospital discharge outcomes (including death, abscondment or transfer to another facility) and date of discharge. The WHO definition of death due to RTA is “death within 30 days of an RTA” [18], but since this was a retrospective study, we, in this paper defined “hospital deaths” as death that occurred at the hospital emergency unit and after admission to a hospital department. Data were also collected on the total number of all patients admitted to emergency units in the three-year period.

Data sources were the registers of the emergency units and hospital departmental wards (surgery, orthopedic and intensive care units) in 20 hospitals of the selected districts. A standardized Excel spreadsheet (version 2016) was used to collect the information from the different registers at police stations and hospitals. The data were collected by a team of 11 trained health professionals, supervised by the principal investigator, between April and August 2018.

### Analysis

Data from the Excel spreadsheet were exported into EpiData (version 2.2.2.182, EpiData Association, Odense, Denmark) for analysis. Results were analyzed using descriptive statistics (proportions, measures of central tendency, and variation). Selected socio-demographic and clinical characteristics were assessed in relation to hospital deaths using the chi square test and results presented as relative risks (RR) along with 95% confidence intervals (CI). The level of significance was set at 5% (*P <* .05).

The trends of RTA and their impacts were also assessed through compiling the existing data across the eight districts and a sequence of interrupted time-series models was estimated, one each for the number of RTA, injuries and death across the study periods: 2015, 2016 and 2017. Segmented ordinary least-squares (OLS) regression were employed using Newey-West standard errors to accommodate for serial auto-correlation [19, 20], and adjusted for any potential effect of seasonality (e.g., rainy versus dry) on the outcomes. The interrupted time-series regression model followed the format:

$$ {\mathsf{Y}}_{\mathsf{t}}={\mathsf{\beta}}_{\mathsf{0}}+{\mathsf{\beta}}_{\mathsf{1}}{\mathsf{T}}_{\mathsf{t}}+{\mathsf{\beta}}_{\mathsf{2}}{\mathsf{X}}_{\mathsf{t}}+{\mathsf{\beta}}_{\mathsf{3}}{\mathsf{X}}_{\mathsf{t}}{\mathsf{T}}_{\mathsf{t}}+{\mathsf{\beta}}_{\mathsf{m}}\mathsf{Season}+{\mathsf{\epsilon}}_{\mathsf{t}} $$

where β_0_ estimates the rate of the indicator of interest per 100,000 population at the beginning of 2015, β_1_ estimates the average monthly change in the indicator rate during 2015, T_t_ is the time in months since the start of the study, β_2_ represents the change in the indicator rate that occurred in 2016 and 2017 (2017 designated by indicator variable X_t_), β_3_ represents the difference between the trend in indicator rate during 2016 and 2017 compared to 2015, β_m_ represents rainy versus dry season, and ϵ_t_ the random error term [20]. Autocorrelation of up to three logs was accommodated within our models [21, 22]. Overall trends across the periods defining 2015, 2016 and 2017 were calculated as follows: linear trend during 2015 = β_1_; and linear trend in 2016 and 2017 = β_1_ + β_3_. Differences were considered statistically significant at *p* < .05.

## Results

### Police station records

**Fig. 1:**
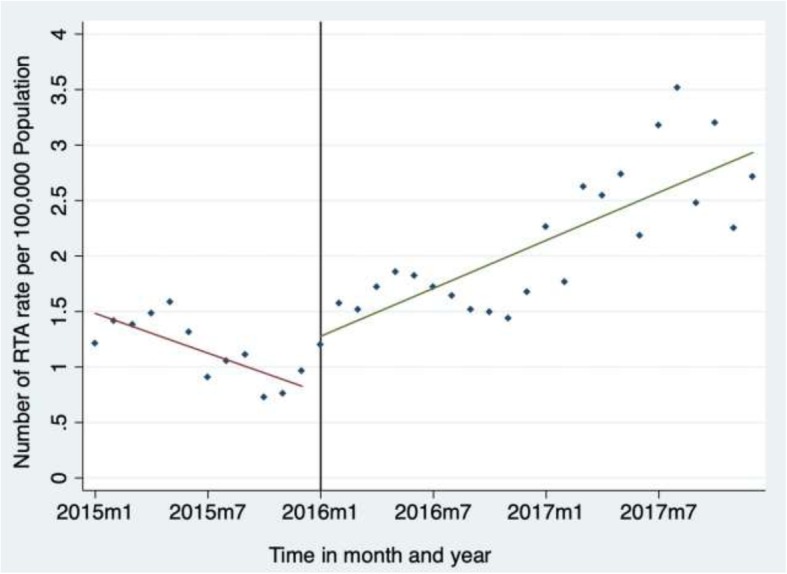
Annual and monthly trends in rates of road traffic accidents (RTA) recorded by police stations in seven districts of Guinea from 2015 to 2017. RTA rates per 100,000 population. Fitted values. Fitted values. m1: *first month (January) of each year (2015, 2016 and 2017).* m7: seventh month (July) of each year (2015, 2016 and 2017)

There was a total of 3,140 RTA with annual and monthly trends shown in Fig. 1. There was an overall annual increase in RTA rates from 14.0 per 100 000 population in 2015, to 19.2 per 100 000 population in 2016 (37.1% annual increase), to 28.7 per 100 000 population in 2017 (49.5% annual increase).

The time-series analysis is shown in Table 1. In the first month of 2015, there were 1.39 RTA cases per 100 000 population (95% CI: 1.21–1.57). During 2015, there was a mean decrease in RTA rate of −.06 per 100 000 population (95% CI: − 0.10, − 0.03) per month. Compared to the trend in RTA rates observed during 2015, the trend in RTA rates in 2016 and 2017 were .14 per 100 000 population higher (95% CI: .10–.19). Overall, the rate of RTA during 2016 and 2017 increased by .07 per 100 000 population on average per month. For injuries, there were .77 RTA victims injured per 100 000 population (95% CI: .66–.88) in January 2015. Over the same year (2015), there was a mean decrease in injury rates of − 0.02 per 100 000 population (95% CI: −.03–.00) per month. Compared to the trend in injury rates during 2015, the trend in injury rates in 2016 and 2017 increased by .07 per 100 000 population (95% CI: .04–.09). Globally, the injury rate during 2016 and 2017 was .05 per 100 000 population (95% CI: .03–.07) higher on average per month. Deaths from RTA showed no statistical difference over the 3 years. There were no associations of RTA trends with season.

**Table 1:** Parameter estimates for monthly RTA, injuries and deaths from 2015 to 2017 in the selected eight urban districts, Guinea from 2015 to 2017

	RTA			Injuries			Deaths		
	β	95% CI	P	β	95% CI	P	β	95% CI	P
Rate of outcome in 2015 (β_0_)	1.39	(1.21; 1.57)	<.001	.77	(.66; .88)	<.001	.13	(.03; .27)	.0460
Average monthly change in rate in 2015 (β_1_)	−.06	(−.10; −.03)	<.001	−.02	(−.03; −.00)	.0470	.01	(−.02; .01)	.3430
Change in rate from 2015 to 2016 and 2017 (β_2_)	−.56	(.19;.93)	.0040	.01	(−.21; .24)	.9120	−.00	(−.07; .02)	.9850
Difference between trend in outcome rate in 2016 and 2017 compared to 2015 (β_3_)	.14	(.10; .19)	<.001	.07	(.04; .09)	<.001	.01	(−.01; .02)	.2800
Overall trends									
*Linear Trend in 2015 Period (β*_*1*_*)*	−.06	(−.10; −.03)	<.001	−.02	(−.03; −.00)	.0470	.01	(−.02; .01)	.3430
*Linear Trend in 2016 and 2017 (β*_*1*_ *+ β*_*3*_*)*	.07	(.05; .09)	<.001	.05	(.03; .07)	<.001	0	(−.00; .00)	.3039

**Fig. 2:**
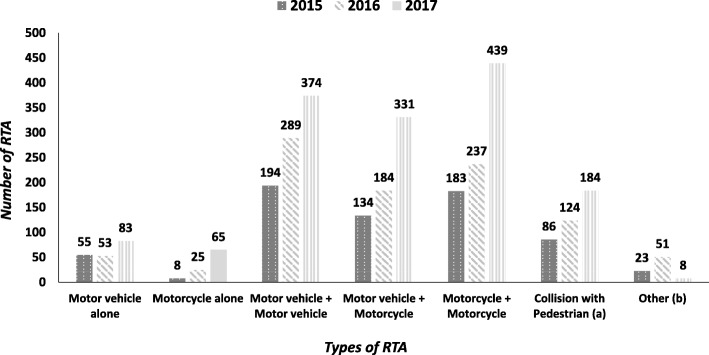
Annual trends in types of road traffic accident (RTA) recorded by police stations in seven districts of Guinea from 2015 to 2017. (a): *vehicle + pedestrian; motorcycle + pedestrian*. (b): *motorcycle + bicycle; motor vehicle + bicycle; bicycle alone; not recorded*. *Note: there were 10 RTA with no information recorded*

For each type of RTA, there was an annual increase from 1 year to the next as shown in Fig. 2**.** The most common type of RTA involved a combination of two vehicles (motor vehicles and/or motor cycles) representing 76% of all RTA cases. This was followed by collisions of a vehicle with pedestrians (13%) and a motor vehicle or motor cycle alone (9%). Motorcycle accidents increased dramatically in the three-year period from 12.0% (2015) to 30.7% (2017).

**Fig. 3:**
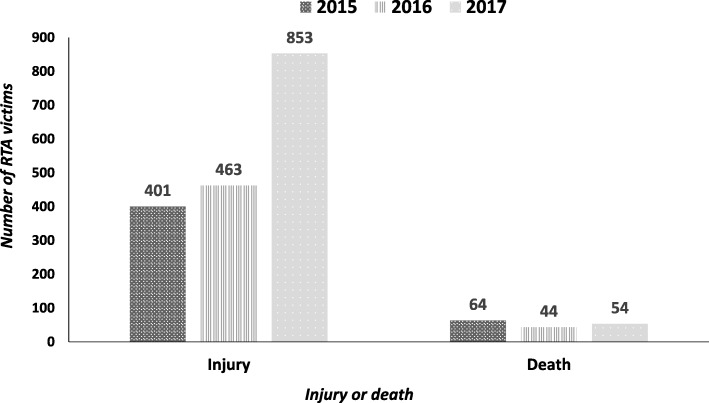
Annual trends in injuries and deaths among road traffic accident (RTA) victims recorded by police stations in seven districts of Guinea from 2015 to 2017. Of the 1717 injuries, 870 (51%) were minor and 847 (49%) were severe based on the opinion of the receiving health facility

In 925 RTA, there was no record of the number of persons involved. In the remaining 2,215 RTA, there were 4,340 persons involved resulting in 40% being injured and 4% being killed (Fig. 3).

### Hospital records

There was a total of 27,751 RTA victims admitted to hospital emergency units: this comprised 22% of the 125,882 persons admitted with all types of illness during the 3 years. The annual rate of RTA victims decreased from 177.3 per 100 000 population in 2015 to 164.5 per 100 000 population in 2016 and then increased at 210.1 per 100 000 population in 2017 (27.7% annual increase).

Socio-demographic characteristics of RTA victims are shown in Table 2. The commonest age group was 15–24-year old (33%) and more males (71%) were victims than females. There was no clear pattern regarding occupation. In two thirds of cases, there was no information about the type of road user involved in the RTA. In the other one third of victims, passengers, motorcyclists and pedestrians were the most affected group.

**Table 2:** Sociodemographic characteristics of road traffic accident victims admitted to hospital emergency units in eight districts of Guinea from 2015 to 2017

Characteristics	N	(%)
Age in years		
<15	4395	(15.8)
15–24	9132	(32.9)
25–34	6855	(24.7)
35–44	3241	(11.7)
45–64	3052	(11)
≥65	913	(3.3)
Not recorded	163	(0.6)
Sex		
Male	19747	(71.2)
Female	7991	(28.8)
Not recorded	13	(0.0)
Occupation		
Students	7151	(25.8)
Work men/women	7120	(25.7)
Farmer/housewife	5186	(18.7)
Sellers	3372	(12.2)
Employees	2513	(9.1)
Children (< 15 and not a student)	1723	(6.2)
Not recorded	686	(2.5)
Type of road user		
Passengers	4626	(16.7)
Motorcyclists	3092	(11.1)
Pedestrians	1129	(4.1)
Drivers	60	(0.2)
Pedal cyclists	56	(0.2)
Not recorded	18788	(67.7)

*RTA* Road traffic accident

Clinical characteristics of RTA victims are shown in Table 3. The large majority of victims presented to hospitals and 86% were managed in the emergency unit without being admitted to hospital wards. The predominant injury involved soft tissue and about 10% of victims sustained a fracture, usually of the lower limbs. The head and lower limbs were the two commonest anatomical sites of injury. Most patients had a normal Glasgow coma score, but 1.9% of all RTA victims had heavy or deep coma.

**Table 3:** Clinical characteristics of road traffic accident victims admitted to hospital emergency units in eight districts of Guinea from 2015 to 2017

Characteristics		N	(%)
Type of hospital	Public hospitals	27348	(98.5)
	Private hospitals	403	(1.5)
Admission to hospital departments	No hospital admission	23240	(83.6)
	Surgery department	2171	(7.8)
	Orthopedic department	546	(2.0)
	Intensive care unit	207	(0.7)
	Neurosurgery department	17	(< 0.1)
	Not recorded	824	(3)
Predominant type of injury	Soft tissue	21160	(76.2)
	Facture	2682	(9.7)
	Joint	377	(1.4)
	Dislocation	295	(1.1)
Main anatomical site of injury	Head	10350	(37.3)
	Lower limb	9179	(33.1)
	Upper limb	5514	(19.9)
	Chest	604	(2.2)
	Abdominal –Lumbar	951	(3.4)
Location of fracture	Lower limb	1770	(6.4)
	Upper limb	764	(2.8)
	Mandible	164	(0.6)
	Ribs	61	(0.2)
Glasgow Coma Score ^a^	Normal	25769	(92.9)
	Mild coma	1185	(4.3)
	Heavy coma	327	(1.2)
	Deep coma	201	(0.7)
	Not recorded	269	(1.0)

*RTA* Road traffic accident, Glasgow coma score- normal = 15; mild coma = 14–10; heavy coma = 9–7; deep coma = 6 = 3

Hospital interventions are shown in Table 4. Skin cleansing and simple bandaging together comprised 69.1% of all interventions, but 13.5% required general surgery (usually minor in nature) and 4% had reductions or fixations of their fractures.

**Table 4:** Medical and surgical interventions on road traffic accident victims admitted to hospital emergency units in eight districts of Guinea from 2015 to 2017

Medical and surgical interventions	N	(%)
Total number of medical and surgical interventions	34832	
Skin cleansing	15393	(44.2)
Simple bandaging	8672	(24.9)
Fracture reduction or fixation	1322	(3.8)
Blood transfusion	28	(< 0.1)
Neurological surgery	17	(< 0.1)
General Surgery	4704	(13.5)
*Minor*	4416	(12.7)
*Complex*	280	(0.8)

**Fig. 4:**
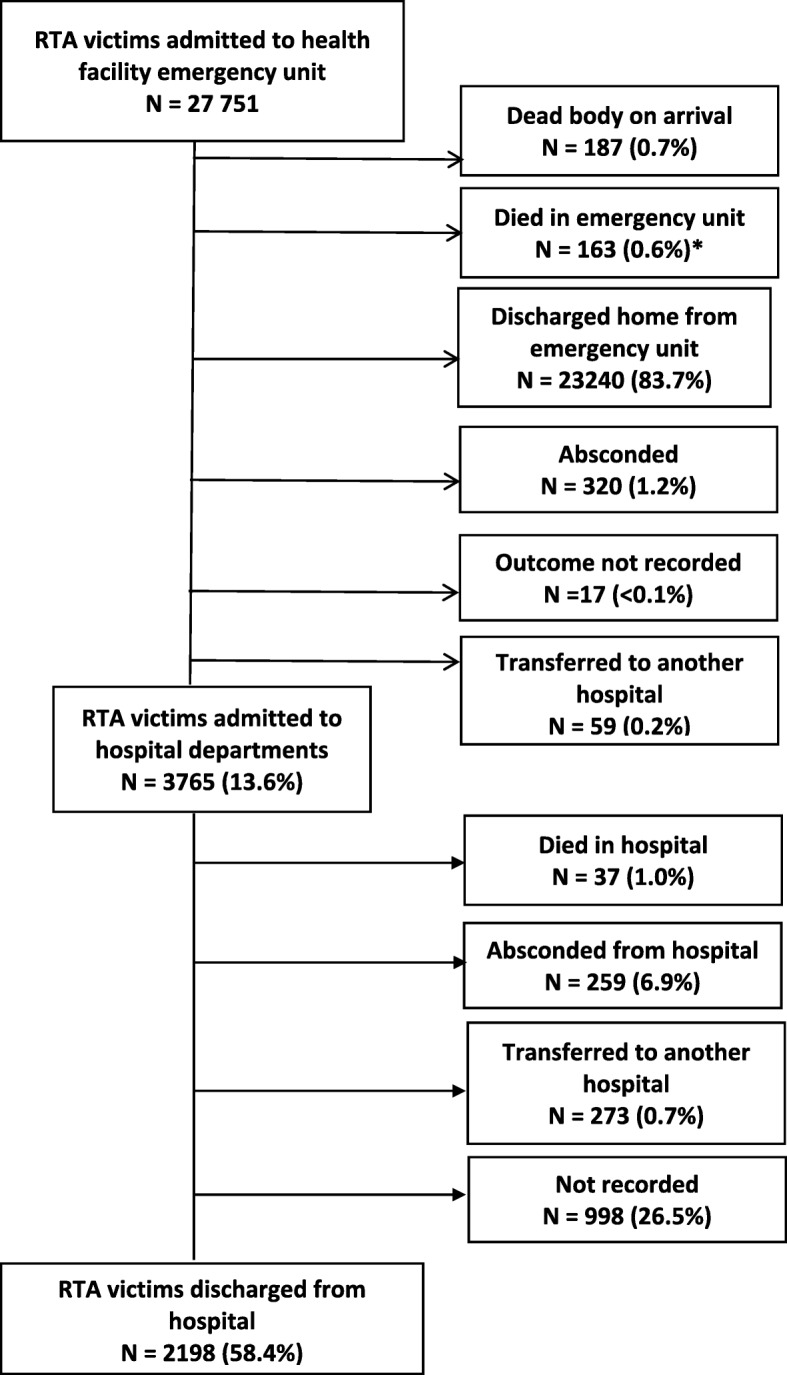
Outcomes of road traffic accident victims presenting to emergency units of health facilities and admitted to hospital in eight districts of Guinea from 2015 to 2017. RTA = road traffic accident. *109 patients died within 24 h and 54 died in the emergency unit but the time was not recorded

Of 27,751 RTA victims, there were 0.7% victims who were dead on arrival and 0.6% who died in the emergency department (all the deaths occurred within 24 h with the time of death recorded in 67% of patients). About 14% of victims were admitted to hospital wards, 84% were discharged home. Of those admitted to hospital wards, about 1% of the victims died. About 58% were discharged home with the remainder absconding from hospital or transferring to another hospital. Also, 27% of the patients had no record about their outcomes (Fig. 4). The median duration of hospital stay was 5 days (IQR = 1–20).

Altogether there were 1.4% deaths of all RTA victims admitted to hospitals. The death rate ranged from 2.4 per 100 000 population in 2015 to 3.7 in 2017. Baseline risk factors for death after admission to hospital are shown in Table 5. Factors associated with death were being male (*p* = .04), being a child under 15 years (*p* = .045) or an elderly person aged ≥65 years (*p* < .001), and having head injury or coma (*p* < .001). The risk of death in relation to lower GCS increased progressively, with 54% of those in heavy or deep coma dying in hospital.

**Table 5:** Selected baseline characteristics associated with risk of death in road traffic accident victims admitted to hospital in eight districts of Guinea from 2015 to 2017

Baseline characteristics		Admitted to hospital	Known death occurring after presentation to a hospital^b^		Crude RR^c^ (95% CI)	*P* value
		N^a^	n	(%)		
Sex:						
	Female	7691	45	(0.59)	Ref	
	Male	18850	155	(0.82)	1.41 (1.01–1.96)	0.04
	Not recorded	8	0			
Age in years	Children (< 15)	4238	40	(0.94	1.43 (1.01–2.02)	0.045
	Adults (15–64)	21321	141	(0.60)	Ref	
	Elderly (≥65)	855	16	(1.87)	2.83 (1.70–4.72)	< 0.001
	Not recorded	135	3	(2.22)		
Type of hospital	Public	26159	197	(0.75)	Ref	
	Private	390	3	(0.77)	1.02 (0.33–3.18)	0.999
Main anatomical site of injury						
Head	No	16739	72	(0.43)	Ref	
	Yes	981	128	(1.31)	3.01 (2.26–4.01)	< 0.001
	Not recorded	9	0			
Chest						
	No	25960	196	(0.76)	Ref	
	Yes	585	3	(0.51)	0.68 (0.22–2.12)	0.717
	Not recorded	4	1			
Abdominal						
	No	26158	196	(0.75)	Ref	
	Yes	389	4	(1.30)	1.37 (0.51–3.67)	0.674
	Not recorded	2	0			
Glasgow Coma Score						
	Normal	24945	75	(0.30)	Ref	
	Mild coma	1134	21	(1.85)	6.16 (3.81–9.96)	< 0.001
	Heavy	286	53	(18.53)	8.45 (5.96–11.98)	< 0.001
	Dep coma	59	21	(35.59)	178.4 (118.5–178.5)	< 0.001
	Not recorded	125	30			

^a^ = 187 RTA victims dead on arrival, 998 RTA victims with unrecorded unit of admission and those who absconded were excluded

^b^ = includes death occurring in hospital emergency unit and after admission to a hospital department

*RR* Relative risk, *CI* Confidence interval

^c^ = Fisher exact test

## Discussion

This is the first study in Guinea to comprehensively examine RTA in eight districts where there have been reports of a high burden of RTA in the past years, using both data from hospitals and police stations. There were three main findings.

First, according to police records, the burden of RTA increased during the three-year study period. Over three quarters of RTA involved two vehicles, which may relate to the growing number of motor vehicles on the road. The rise in the rate of RTA cases was accompanied by a similar rise in the rate of RTA victims. These findings are in line with previous studies from other African countries [6, 7, 10].

Even though we did not assess why the burden of RTA has increased, there might be several possible reasons. First, improvements in socio-economic standards and the production of more affordable second-hand vehicles imported from European countries and motorcycles imported from India and China have led to a growing number of vehicles on the roads. Road safety legislation, road infrastructure and affordable ways to maintain vehicles have not kept pace with the surge in vehicle numbers. Studies in Gambia, Burkina Faso and Tanzania have shown that many injuries occur at hot spots such as road intersections with or without traffic lights, during rush hours and at night [23–25], and these factors may play a part in Guinea. Human behavior also plays an important role with speeding, careless driving and driving under the influence of alcohol being important factors contributing to RTA in many countries, and probably also in Guinea [26, 27].

Second, according to police records, 40% of RTA victims were injured and were referred to hospital. These numbers were much lower than the numbers admitted to emergency units according to hospital records. Reasons for the observed discrepancies are speculative but include lack of information about injuries in police records and self-reporting of RTA victims to hospital without involving the police. From hospital records, over 27,000 RTA victims were admitted to emergency units during the study period. This resulted in an increased workload for hospitals requiring more staff and resources. The fact that most RTA victims presenting to hospital emergency units were discharged home and did not require admission to a hospital ward contrast with some studies reporting higher RTA-related morbidity and mortality [27, 28]. This discrepancy may be explained by the fact that those RTA victims who were discharged home had minor injuries such as scratches or slight confusion. However, our findings are similar to those conducted in Ethiopia and Gambia which found low rates of hospital admission and deaths [11, 23].

Males and young adults were predominant among the RTA victims which concurs with studies from everywhere including industrialised countries [23, 25, 29–31]. The reasons that male and young adults are predominant are several. It relates to their inexperience [32] and the fact that they are a very active group on the roads, i.e. they drive more than other groups. Furthermore, higher exposure (mileage) together with psychological factors (sensation seeking, peer influence, driving under the influence of alcohol) make them a risky group [33, 34]. Injuries to the head and limbs and soft tissue trauma were the predominant types of injury, confirming findings from a study in Gambia [23].

Third, serious injury resulted in death but altogether just over 1% of all RTA victims died. This mortality may be underestimated as a quarter or more of patients admitted to hospital had no record of their outcome. In addition, we could not document the outcomes in those transferred to hospitals other than those included in the study. The case-fatality rate observed in our study was therefore lower than what has been reported in other studies [7, 28, 35, 36]. However, it was similar to the general low morbidity and mortality rates reported in Ethiopia and Gambia [11, 23]. According to the police records, the numbers of fatal RTA at the scene of the accident remained the same over the 3 years. These findings, therefore, call for urgent attention to address this growing health threat by putting in place a solid surveillance system through enforcement of the police stations’ capacity to control road traffic, improve the design of roads and keep in place periodic education of people to prevent RTA fatality and guarantee the safety of road users.

In hospital records, nearly half of the deaths occurred at the site of the RTA with the remainder occurring within 24 h. Risk factors for death in those admitted to hospital included being male, a child or an elderly person, having an injury involving the head and being in coma with progressively lower GCS predicting higher probabilities of death. These findings are similar to previous reports on the subject [37].

This study had several strengths. First, the selection of eight urban districts as study sites meant that we covered 45% of the national population, and our findings are probably representative of what is happening in Guinea. Second, we collected data from police stations and hospitals, and while these could not be linked, they nevertheless provide a more holistic picture of RTA in Guinea. Such an approach of using different data sources has been recently recommended as a way of improving data collection and completeness of information about road traffic injury [37]. Third, hospital data were individual-based and thus provided information on RTA victims from admission to emergency units to their outcomes. Finally, the study was conducted and reported in line with the Strengthening the Reporting of Observational Studies in Epidemiology (STROBE) [38].

However, there were some limitations. Some information in the police station records were missing, such as numbers of injured persons and in hospital records, such as details about road users. Valuable information in police records was also not regularly captured such as whether 4-wheel vehicles involved in RTA were cars, taxis, buses or trucks, the potential cause of the accident such as speeding and/or drunk-driving and whether safety belts or crash helmets were worn by drivers and passengers.

There are a number of implications and recommendations from this study. First, there needs to be a serious attempt to improve the recording practices in both police and hospital records, especially about potential causes of RTA, the number of people involved, the type of motor vehicle and the time taken to get victims to hospital. There also needs to be better oversight so that unrecorded outcomes are minimized. In parts of the country where the burden of RTA is high, consideration should be given to using and linking electronic records in both police station and hospital sites. This would facilitate better tracking of RTA and victims from the site of the accident to hospital and to an eventual outcome.

Second, with the increasing numbers of RTA and their victims, the country is not on track to halve RTA by 2020 as pledged in the SDGs [4]. What can be done quickly to reduce the numbers of RTA? Geographic information systems have been set up in Tanzania to identify hot spots of RTA [25], and Guinea should consider setting up similar systems. The country also needs to enforce road safety legislation about drink-driving and speeding, improve infrastructure through more paved and better designed roads, roadway lighting and roundabouts and tighten up measures around vehicle safety [26]. Moreover, police stations and hospitals should consider using a checklist when recording RTA information to capture data about availability of first-aid, easy access to hospital and causes of RTA such as human attitudes [16]. Community-based awareness campaigns might also help [39].

Finally, attention needs to be given to reducing the risk of serious injury and death. The most successful interventions to prevent RTA death are speed and drink-driving enforcement [26]. However, RTA deaths can also be reduced by the availability of fast and reliable emergency services in rural areas, the use of telemetric systems including automated accident notification systems connected to emergency services and post traumatic rehabilitation services to prevent complications [40, 41]. These interventions should be prioritized at all levels along with bicycle and motorcycle helmet use and seat-belt use for front and rear passengers.

## Conclusions

Between 2015 and 2017, there was an increase in RTA and numbers of RTA victims in Guinea. The burden on hospitals was high with over 20% of emergency unit admissions being due to RTA. The majority of victims admitted to emergency units were discharged home, but nearly 15% required hospital ward admission. Altogether, there was a low proportion of deaths but about half of the deaths occurred before hospital admission and most of the remainder occurred in the emergency unit and within 24 h. Risk factors for death in those admitted to hospital included being male, a child or elderly person and having head injury and coma. More needs to be done in Guinea to reduce RTA and prevent serious injury and death.

## Data Availability

All data generated or analyzed during this study are available from the corresponding author on reasonable request.

## Abbreviations

CI: Confidence Intervals
GCS: Glasgow Coma Score
IQR: Inter Quartile Range
OLS: Ordinary Least-Squares
RR: Relative Risk
RTA: Road traffic accidents
SDG: Sustainable Development Goals
STROBE: Strengthening the Reporting of Observational Studies in Epidemiology
WHO: The World Health Organization
